# Sex in Immune Cells and Parasitic Diseases — A Complex Relationship

**DOI:** 10.1111/imr.70097

**Published:** 2026-01-16

**Authors:** Barbara Honecker, Charlotte Sophie Hansen, Hanna Lotter

**Affiliations:** ^1^ Bernhard Nocht Institute for Tropical Medicine Hamburg Germany

**Keywords:** immune response, parasitic diseases, sex differences, sex hormones

## Abstract

Epidemiological studies consistently show that many parasitic diseases affect males more frequently than females. These disparities are multifactorial, arising partly from gender‐specific behaviors that influence exposure risk and health‐seeking practices, especially in low‐ and middle‐income countries. Increasing evidence also highlights that biological sex differences within the immune system significantly shape susceptibility to and control of parasitic infections. Recent advances combining classical immunology with single‐cell transcriptomics have revealed hormonal and chromosomal factors driving sex‐specific differences in innate and adaptive immune cells. These differences can critically influence the course and outcome of parasitic diseases. However, many studies on parasitic diseases still lack adequately sex‐disaggregated data or fail to apply state‐of‐the‐art immunological analyses needed to fully characterize biological sex effects. Studies in rodent models that mirror the sex bias observed in humans provide valuable tools to analyze immune mechanisms at the cellular level and dissect underlying biological differences. In this review, we summarize current knowledge on sex differences in key cellular components of innate and adaptive immunity and discuss their relevance for selected parasitic diseases of major global importance—leishmaniasis, Chagas disease, amebiasis, schistosomiasis, and malaria.

## 1 Introduction

Sex‐based differences in susceptibility, immune responses, and outcomes of infectious diseases are well documented [1, 2]. While such differences have been extensively characterized in bacterial and viral infections [3, 4, 5], their significance in parasitic diseases remains comparatively underexplored. This is notable given that parasitic infections continue to represent a major global health burden, affecting millions of individuals and contributing substantially to morbidity and mortality worldwide [6]. The impact of these diseases is further amplified by the complex nature of host–parasite interactions, which influence transmission dynamics, disease severity, and the effectiveness of prevention and control strategies [7, 8].

Parasitic diseases, many of which are classified as neglected tropical diseases (NTDs) [9], disproportionately affect populations in low‐ and middle‐income countries. In these settings, poverty, including lack of sanitation, access to drinking water and health care, as well as gender‐specific behaviors, sustain transmission cycles [10] and complicate the identification of intrinsic biological sex differences. Moreover, research on tropical infectious diseases faces persistent challenges due to limited funding [11, 12], which constrains access to high‐quality biological samples [13], specialized laboratory infrastructure, and the development of costly animal models [14]. These limitations hinder efforts to unravel how biological sex influences disease susceptibility, progression, and therapeutic outcomes, leaving many sex‐specific mechanisms insufficiently characterized.

Current evidence from the literature indicates a pronounced male bias in both infection rates and disease severity across numerous parasitic infections [1, 15, 16, 17, 18, 19]. Although the precise immunological mechanisms underlying these sex‐specific differences are not yet fully elucidated, growing evidence suggests that both sex hormones and chromosomal differences between males and females play crucial roles in shaping host immune responses [2, 20]. In recent years, a surge of studies investigating sex‐based immunological variation has profoundly expanded our understanding of immune dimorphism. This emerging body of work spans from broad distinctions in immune cell populations between the sexes to more refined insights into how sex hormones modulate immune cell function at the single‐cell level [21, 22, 23, 24, 25]. Such advances promise to consolidate existing findings and enhance our capacity to identify overarching mechanisms governing sex‐specific immunity across diverse parasitic diseases.

This review explores how biological sex shapes innate and adaptive immune cell function in the context of infection, integrating evidence from human epidemiological and immunological studies. Sex‐specific immune mechanisms in selected parasitic diseases are illustrated herein as representative models for understanding fundamental male/female differences in parasitic infection biology.

## 2 Role of Gender in Parasitic Infections

In contrast to biological characteristics such as reproductive organs, hormones, and chromosomes, gender refers to the social and cultural dimensions of identity that shape behaviors, roles, and access to resources [26]. These gendered factors can significantly influence exposure to parasitic diseases and access to healthcare [27, 28]. This is particularly evident in low‐ and middle‐income regions, where the disease burden from parasitic diseases is particularly high [29, 30]. For instance, men are often more exposed to the parasites causing schistosomiasis through fishing or irrigation, while women encounter contaminated water during domestic chores such as laundry and bathing [31, 32]. Gender norms also affect health‐seeking behavior in that women may face mobility and financial barriers, whereas men may delay treatment due to social expectations of resilience [33]. Moreover, many disease control programs remain gender‐blind, overlooking differences in participation and accessibility. Integrating gender‐responsive approaches, such as flexible treatment delivery and targeted communication, improves intervention outcomes [28]. Despite these findings, many studies on sex differences in parasitic diseases still do not disaggregate between sex and gender, hindering the identification of sex‐ in contrast to gender‐related effects on infection. In addition, sufficient resources to investigate underlying immunological mechanisms are not always available [34].

While gender profoundly shapes the social contexts through which parasitic diseases are transmitted and managed, biological sex determines the physiological susceptibility to disease progression or control.

## 3 Chromosomes and Hormones in Biological Sex Differences

Sex assigned at birth is determined by the interplay of chromosomal sex (XY in males, XX in females), gonadal differentiation (testes or ovaries), and circulating sex hormones (androgens, estrogens, and progesterone), which together guide sexual development and influence immune function throughout life [35]. The X chromosome encodes about 830 protein‐coding genes, many directly involved in immune regulation, including *forkhead box protein P3* (*FOXP3*), *CD40LG*, *toll‐like receptor* (*TLR*)*7*, *CXCR3*, and *IL2RG* [36, 37]. In contrast, the Y chromosome comprises around 106 genes, and whether any of them directly influence immune functions remains unclear [38, 39].

To balance gene dosage, one X chromosome in individuals with more than one X undergoes X‐chromosomal inactivation (XCI). This process involves the coating of the future inactive X chromosome by the long non‐coding RNA Xist, which renders the chromosome largely inaccessible to the transcriptional machinery. However, up to 30% of X‐linked genes can escape XCI in a tissue‐specific manner [37, 40], leading to elevated expression of immune genes such as *TLR7* and contributing to stronger immune activation in females [41]. Beyond gene dosage, both the inactive X chromosome (Xi) and the Y chromosome modulate autosomal and active X (Xa) gene expression in a cell type‐specific manner [42]. With increasing age, mosaic loss of sex chromosomes occurs. In females, this loss predominantly affects the inactivated X chromosome [43, 44] and its immunological consequences remain largely unexplored. In males, age‐related loss of the Y chromosome alters the transcription of hundreds of autosomal genes in leukocytes, demonstrating the regulatory influence of Y‐linked elements [45]. Roughly 37.5% of human protein‐coding genes show sex‐differential expression in at least one tissue, with the majority (96%) of autosomal origin [46]. This widespread autosomal involvement indicates that sex‐related transcriptional differences are a genome‐wide phenomenon rather than being restricted to the sex chromosomes.

Complementing chromosomal influences, sex hormones further shape immune function through diverse signaling pathways. Females and males differ markedly in sex hormone production and circulating levels [47], with estrogens and progesterone predominating in females, and androgens, such as testosterone and dihydrotestosterone (DHT), in males. Testosterone gradually declines with age in males, while estrogen and progesterone fluctuate during the menstrual cycle in female reproductive years and drop sharply after menopause, influencing immune function, vaccine efficacy, and disease susceptibility [48, 49]. The ovaries and testes represent the main sources of sex hormones, although steroidogenesis can also occur in peripheral tissues [47].

Sex hormones influence cellular functions through two routes: a genomic pathway mediating long‐term effects and a non‐genomic pathway triggering rapid responses. In the genomic pathway, estrogens, androgens, and progesterone bind to their intracellular receptors (estrogen receptor α/β (ERα/ERβ), androgen receptor (AR), progesterone receptor (PR)), which translocate to the nucleus and interact with hormone‐response elements (HREs; e.g., ERE, ARE, PRE) to regulate gene transcription [18, 48]. In contrast, non‐genomic signaling occurs when sex hormones engage membrane‐bound receptors, activating kinase cascades such as phosphoinositide 3‐kinase/protein kinase B (PI3K/Akt) and mitogen‐activated protein kinase/extracellular signal‐regulated kinase (ERK/MAPK). These pathways phosphorylate transcription factors and thereby modulate gene expression indirectly [48, 50]. Through these signaling cascades, estrogens generally enhance, whereas androgens tend to suppress immune responses. Their specific effects depend on cell type, tissue environment, hormonal milieu, and disease state, which will be discussed in the subsequent sections in the context of parasitic infections.

## 4 Influence of Biological Sex on Immune Cells

### 4.1 Sex Differences in Cells of Innate Immunity

Innate immune cells are present in high numbers in the peripheral circulation and can be rapidly mobilized to sites of infection. By acting within the first hours of pathogen encounter, they play a central role in initiating host defense and shaping the early inflammatory milieu. Emerging evidence highlights substantial sex‐based differences in several innate immune cell populations, encompassing cell frequency, activation potential, and effector function. These differences have potential implications for infection susceptibility, immune‐mediated pathology, and therapeutic responsiveness. In the following sections, we discuss selected examples of innate immune cell types (monocytes, macrophages, neutrophils, NK, and NKT cells) that exhibit pronounced sex differences and are implicated in immune regulation of parasitic diseases.

#### 4.1.1 Monocytes and Macrophages

Monocytes are essential in early immunity against infections and constitute approximately 10%–20% of peripheral blood mononuclear cells (PBMCs) [51]. Even at steady state, healthy individuals display sex‐specific differences in the composition of peripheral monocytes, which comprise three major subsets [52]. Classical CD14^+^CD16^−^ pro‐inflammatory monocytes appear to occur at higher frequencies in males than in females, at least among Caucasian populations [2, 53, 54, 55, 56]. Conversely, some studies have reported that non‐classical CD14^−^CD16^+^ monocytes, characterized by anti‐inflammatory properties, are more prevalent in females [55, 57]. No consistent sex differences have been reported for intermediate CD14^+^CD16^+^ monocytes [55, 57, 58]. The total percentage of monocytes increases with age in both sexes [23, 57]. These three monocyte subsets have distinct functional profiles. Classical monocytes mediate innate immune sensing and early inflammatory responses by producing pro‐inflammatory cytokines (e.g., IL‐1β, IL‐6, IL‐8, TNF) and chemokines (e.g., CCL2, CCL3, CCL5, CXCL1), engaging in phagocytosis, generating reactive oxygen species (ROS), and differentiating into monocyte‐derived macrophages (moMΦs) and dendritic cells (moDCs). Intermediate monocytes, the smallest subset, are primarily involved in antigen presentation, cytokine secretion, and apoptosis regulation. Non‐classical monocytes contribute to complement‐ and Fcγ receptor‐mediated phagocytosis as well as vascular adhesion [52, 58, 59, 60, 61]. Differentiation of monocytes into moMΦs occurs upon migration of monocytes into tissues, primarily during inflammatory processes, and has distinct functions from tissue‐resident macrophages that arise during embryonic development [59].

Sex‐related functional differences are evident in several processes in monocytes and macrophages. In murine macrophages, interferon (IFN)‐stimulated genes (ISGs) were grouped into functional clusters: RNA processing, antiviral effectors, metabolic regulation, and inflammatory mediators [62, 63]. Expression was generally higher in females, with the antiviral cluster showing the largest increase [62]. Analysis of these ISGs in human monocytes confirmed higher baseline IFN‐responsive gene expression in females, particularly in pathways related to cytokine signaling (e.g., *IFIT1*, *IFIT3*, *MX1*, *STAT1*) and monocyte‐mediated PI3K signaling in B cells (e.g., *CD40*, *CD79A*, *PIK3CG*) [22]. This dedicated IFN response signature (e.g., *Ifi27*, *Irf1*, *Irf7*, *Irf9*, *Isg20*) is also higher in females in mice [21].

On the protein level, classical monocytes from males produce higher amounts of TNF, IL‐6, IL‐1β, and IL‐12, as well as the chemokines CCL2 and CXCL1 [54, 58, 64, 65] and express higher amounts of CCR2, the receptor for CCL2 [58, 66]. Ex vivo treatment of human monocytes with androgens in the context of stimulation with parasitic lysate slightly enhances TNF and significantly increases CXCL1 production, but does not affect CCL2 production [58]. Longitudinal systems‐level analysis in transgender men receiving testosterone during gender‐affirming hormone therapy (GAHT) showed enhanced monocyte responses to lipopolysaccharide (LPS) stimulation. This effect was characterized by increased expression of *TNF, IL‐1, IL‐6*, and the NFκB pathway component *NFKB*, along with upregulation of the surface receptor *SLAMF7*. Notably, these changes were accompanied by an attenuation of type I IFN signaling following ex vivo stimulation with LPS as well as TLR7/8 agonists [25]. LPS stimulation reduces autophagy only in monocytes from males, whereas in females, it results in increased chemotaxis and decreased ERα expression [65]. These findings were corroborated in mice, where castration or testosterone replacement modulated classical Ly6C^hi^ monocytes in the context of infection, particularly influencing TNF and CXCL1 production [58]. Also in murine macrophages, TNF release during wound healing was shown to be enhanced by testosterone via AR signaling [67]. Among tissue‐resident macrophages in the peritoneum of mice, cells from females express more *Tlr2, Tlr3, Tlr4*, and *Myd88*, exhibit enhanced phagocytosis, and more efficient bacterial killing during peritonitis due to higher NADPH oxidase activity [68]. Additionally, during steady state in murine macrophages, testosterone treatment reduces TLR4 expression, whereas estrogen treatment increases TLR4 expression and elevates serum TNF levels [69, 70]. Similar to monocytes, adipose tissue macrophages of male mice express higher levels of *Cx3cr1*, *Cxcl1, Cxcl2, Ccl2*, and *Ccl3*, among others, indicating a more pronounced migratory phenotype [71].

In response to different cytokine milieus, macrophages may polarize into classically activated M1 macrophages, which are generally considered pro‐inflammatory and aid in clearing pathogens, and alternatively activated M2 macrophages with an anti‐inflammatory phenotype that contribute to tissue repair [72]. In comparison with females, bone marrow‐derived macrophages (BMDMs) of male mice display a more polarized phenotype regarding both M1 and M2 polarization, higher M1‐associated ROS production and an overall stronger inflammatory response to LPS [73]. BMDM polarization has been shown to be mediated by sex hormones, with estradiol reducing production of IL‐1β and promoting an anti‐inflammatory phenotype [74]. During bacterial pneumonia in mice, infiltration of both monocytes and macrophages into the lung is higher in males than females, alongside a stronger M1 macrophage phenotype characterized by higher expression of inducible nitric oxide synthase (iNOS) and CD80 in males compared with females [75].

In summary, there are sex‐specific differences in monocyte subset frequencies and their functional responses, with higher amounts of classical monocytes and a more pro‐inflammatory phenotype in males, while IFN‐response signatures are more pronounced in monocytes from females. The enhanced pro‐inflammatory phenotype and elevated predisposition to migration in males are conserved in macrophages, which furthermore exhibit stronger polarization than macrophages from females. These differences are shaped in part by sex hormones and may influence susceptibility to parasitic diseases.

#### 4.1.2 Neutrophils

A growing body of evidence reveals substantial sex‐based differences in neutrophil abundance, maturation, signaling, and effector functions. Neutrophilic granulocytes are the most abundant leukocyte subset in human blood, comprising approximately 50%–70% of circulating white blood cells, with a tendency to higher counts in males [54]. In contrast, sex differences in eosinophils (2.3%) or basophils (0.4%) are not reported in healthy individuals, though differences may emerge in the context of specific diseases [76, 77] such as the murine asthma models [78]. Peripheral neutrophils exhibit sex‐specific differences in maturation that influence their activation potential. In young healthy adults (20–30 years), neutrophils from males display a pronounced immature neutrophil gene signature compared to age‐matched females, resulting in a less activated phenotype characterized by surface marker expression and reduced responsiveness to cytokine stimulation [79]. In females, distinct neutrophil clusters are enriched in type I ISGs, driving stronger responses to specific TLR ligands such as the TLR7/8 agonist R848 [80]. Studies in men with Klinefelter syndrome (XXY) and in prepubescent children indicate that these differences are hormone‐driven rather than due to variance in X chromosome dosage [80]. Single‐cell RNA sequencing of neutrophils from healthy donors identified five subsets with sex‐specific profiles: females showed higher type I ISG expression and a less inflammatory signature, while males exhibited more inflammatory and chemotactic profiles [24]. Mouse studies support this, with naive female bone marrow neutrophils expressing higher amounts of type I ISGs, linking maturation status to IFN responsiveness [81].

Sex differences also extend to neutrophil migration. Higher chemokine levels in males, such as CXCL5 and CXCL6, likely drive increased neutrophil motility [82], while female‐derived neutrophils are less prone to repelled movement. This difference is dependent on regulation of cellular migration by Rho‐associated kinases or Cdc42, as their inhibition leads to migration of neutrophils from males away from signals, whereas neutrophils from females move toward them [83]. These behavioral distinctions extend to acute inflammation, with male neutrophils producing more pro‐migratory prostaglandins, particularly PGE2, likely due to greater availability of the precursor arachidonic acid and the prostaglandin‐synthesizing enzyme COX‐2 [84].

Sex‐specific bioenergetic differences are also evident. Neutrophils from males have higher oxygen consumption rates and extracellular acidification rates, reflecting increased mitochondrial respiration and glycolysis, which are decreased by estradiol treatment [80]. Androgens exert well‐documented effects on neutrophil biology. They stimulate both mature neutrophils and precursors in vitro [85] and accelerate neutrophil recovery in vivo after immunosuppressive therapy in a testosterone dose‐dependent manner [86, 87], an effect also observed in female mice [81]. However, this androgen‐driven mobilization can impair antimicrobial function by promoting the release of immature neutrophils from the bone marrow [88]. Moreover, androgens further modulate immune regulation by reducing degranulation, phagocytosis, and myeloperoxidase activity, while enhancing ROS production and the expression of anti‐inflammatory cytokines such as transforming growth factor (TGF) β1 and IL‐10 [88, 89, 90]. Spontaneous apoptosis occurs more frequently in neutrophils from males than in females [91, 92], whereas estradiol and progesterone delay apoptosis in both sexes and increase intracellular reactive oxygen intermediate production in females [91]. Neutrophils from males respond more robustly to LPS and IFNγ, producing higher levels of TNF, activating MAPK and PI3K pathways, and expressing increased TLR4 levels [93], although a direct androgen dependence still remains uncertain, unlike in monocytes [25, 58].

Neutrophil extracellular trap (NET) formation is essential for pathogen capture and clearance, and shows marked sex‐related differences. Baseline NET release is intrinsically higher in female‐ than male‐derived neutrophils, with age‐ and pregnancy‐associated variations, and is further amplified by LPS stimulation [79, 94]. Estradiol suppresses LPS‐induced NET release in male neutrophils but enhances it in females, whereas testosterone has little effect in males yet promotes NET formation in females [94], highlighting sex hormones as key modulators of neutrophil function.

Taken together, sex profoundly shapes neutrophil abundance, maturation, signaling, effector functions, and migration. Whereas female‐derived neutrophils exhibit stronger type I IFN signatures, male‐derived neutrophils are generally more inflammatory and motile, which may be implicated in sex differences during infections with neutrophil‐dependent immune modulation.

#### 4.1.3 NK Cells

Natural Killer (NK) cells, belonging to group I innate lymphoid cells (ILCs), constitute 5%–20% of circulating lymphocytes and comprise two main subsets, CD56^bright^ cells, which are more regulatory, and the more abundant CD56^dim^ cells, which exhibit stronger cytolytic activity [95, 96].

Males have been shown to possess a higher percentage of NK cells compared to females [2, 23, 97]. Cytolytic CD56^dim^ NK cells increase markedly with age in both sexes [98], with sex‐specific differences evident at the single‐cell transcriptomic level [23]. The increased NK cell numbers in males, accompanied by reduced NK cell activity, are independent of sex hormones, as these differences persist after gonadectomy in mice [97]. Instead, they are linked to lower expression of the X‐linked gene *Kdm6a* in males, which encodes the epigenetic regulator UTX and escapes XCI in females both in humans and mice [97]. NK cell‐intrinsic UTX deficiency in female mice increases the number of NK cells and reduces their IFNγ production, establishing a link between higher UTX expression and the female NK cell phenotype [97]. However, this may change with aging, as transcriptional analyses at the single human NK cell level have shown increased expression of genes enriched in NK‐mediated cytotoxicity and immune effector processes, particularly those involved in the IFNγ‐mediated signaling pathway in aged males [23]. NK cells are highly responsive to type I IFNs, which drive their activation and degranulation, enabling effective clearance of intracellular infections, particularly viral infections. Consistent with this, NK cells display stronger activation in response to TLR7/8‐stimulated female‐derived PBMCs or plasmacytoid dendritic cells (pDCs), a finding linked to higher IFNα production by female‐derived pDCs and reflecting an extrinsic, type I IFN‐mediated effect. Moreover, female‐derived NK cells are intrinsically more responsive to IFNα stimulation, suggesting enhanced IFNα signaling in females [99]. Evidence from animal studies has further highlighted complex, and at times opposing, effects of sex hormones on NK cells. Estradiol has been reported to enhance IFNγ production via classical ERs [100], yet also to suppress NK cytotoxicity by reducing the release of effector molecules such as granzyme B and FasL [101]. In contrast, testosterone treatment during GAHT in transgender men increases IFNγ production in NK cells and enhances responsiveness to monocyte‐derived IL‐12 by upregulation of *IL12RB1* and *IL12RB2* [25]. In vitro DHT treatment experiments demonstrate that this effect is mediated specifically through AR signaling and not affected by inhibition of ER signaling [25].

Overall, NK cell function is shaped by both genetic and hormonal influences, with higher NK cell numbers in males and sex‐specific differences in responsiveness that may contribute to divergent outcomes in the control of intracellular infections. The seemingly contrasting effects of estrogen and testosterone underscore the complexity of hormone–NK cell interactions and highlight the need for further work to disentangle their context‐dependent roles.

#### 4.1.4 NKT Cells

Natural killer T (NKT) cells are a heterogeneous group of non‐conventional T cells expressing both NK and T cell markers, bridging innate and adaptive immunity [102, 103]. They are divided into two subtypes: type I invariant NKT (iNKT) cells, which express a semi‐invariant T cell receptor (TCR), and type II NKT cells, which exhibit a diverse TCR repertoire [104]. Based on CD4 and CD8 expression, NKT cells are further classified into CD4^+^, CD8^+^, and double‐negative (DN; CD4^−^CD8^−^) subpopulations [105]. Unlike conventional T cells that recognize peptide antigens via MHC I/II, NKT cells detect lipid antigens presented by the non‐classical MHC I molecule CD1d and respond to both self and microbial antigens [102]. Activated NKT cells produce diverse pro‐ and anti‐inflammatory cytokines, including IFNγ, TNF, IL‐2, IL‐4, IL‐17, and TGFβ [106]. The strongest known NKT cell activator is α‐galactosylceramide (αGalCer), a lipid molecule originally isolated from a marine sponge [102], though various microbial glycolipid antigens, such as from *Entamoeba histolytica* , can also activate NKT cells via CD1d [107, 108]. NKT cells are rare, comprising < 0.1% of PBMCs [109, 110], though higher frequencies are observed in tissues, particularly the liver [111, 112]. In the murine liver, NKTs account for 20%–30% of hepatic lymphocytes, whereas in humans, they represent < 1% [113]. Total NKT, DN and iNKT cell frequencies also exhibit sex differences, being more abundant in women [110, 114, 115]. Ex vivo, human NKT cell responses are dominated by the chemokines CCL3 and CCL4 and Th1 cytokines IFNγ and TNF, with lower levels of IL‐2, IL‐4, and perforin, and negligible IL‐5, IL‐6, IL‐10, IL‐13, or IL‐17 [116]. αGalCer induces higher intracellular IFNγ, IL‐4, IL‐17, and TNF production in female CD4^+^ and DN NKT cells [110]. In vivo, αGalCer stimulation generates higher serum IFNγ in female mice, a response dependent on estrogen signaling, while testosterone modulates NKT cell activation in males [108, 117, 118].

In summary, NKT cells show pronounced sex‐specific differences, with higher numbers and stronger activation in females, particularly higher release of cytokines like IFNγ. This female bias is driven by estrogen and modulated by testosterone, underscoring the critical influence of sex hormones on NKT‐mediated immunity and disease outcome in the NKT cell‐enriched liver.

### 4.2 Sex Differences in Cells of Adaptive Immunity

Adaptive immune cells, including T and B lymphocytes, are essential for mounting a specific and long‐lasting immune response to pathogens as they undergo clonal expansion and differentiation upon antigen encounter, enabling targeted and memory‐driven immunity. Increasing evidence reveals marked sex‐based differences in adaptive immune cell subsets, affecting their frequency, activation thresholds, cytokine production, and antibody responses. These variations contribute to differences in infection outcomes, vaccine efficacy, and susceptibility to autoimmune diseases between males and females. In the following sections, we highlight key adaptive immune cell populations (B cells, CD8^+^ T cells, CD4^+^ T cells, and regulatory T cells) that demonstrate significant sex‐related disparities, exploring the molecular mechanisms and immunological implications of these distinctions.

#### 4.2.1 B Cells

B cells are a crucial component of the adaptive immune system, responsible for producing antigen‐specific antibodies that neutralize pathogens and facilitate their clearance. Beyond antibody secretion, B cells also present antigens and secrete cytokines, contributing to the regulation of immune responses. In adults, B cells comprise around 10% of PBMCs, with approximately 60% being naïve and 40% memory cells [119, 120]. Naïve and memory subsets include transitional B cells and plasmablasts—the latter being short‐lived antibody‐producing effectors, while bone marrow plasma cells sustain long‐term humoral immunity [121].

Sex‐related differences in B cell biology are well documented. Multiple studies report higher numbers of circulating CD19^+^ B cells and plasma cells in females, particularly from puberty onward [2, 23, 122]. Additionally, CD19^+^CD27^+^ memory B cells increase after puberty in women compared with males, a difference that wanes after menopause and depends on both estrogen and an XX background [123]. B cells express *ESR2* encoding for ERβ and the enrichment of *ESR2*‐binding sites in class‐switch genes indicates a direct hormonal regulation. In a gender‐diverse cohort, estrogen blockade in transgender men reduced class‐switched memory B cells, whereas estradiol treatment in transgender women had no effect. In postmenopausal cisgender women, hormone replacement therapy (HRT) was associated with higher levels of class‐switched memory B cells, while no sex differences were observed before puberty [123]. In addition, single‐cell transcriptomics show stronger B cell‐related signaling in young females compared to males, with activity decreasing with age. In older adults, males exhibit higher expression of inflammatory and senescence‐associated genes, whereas B cell markers remain enriched in females [23].

Sex differences in antibody subclasses reveal that females typically produce higher levels of certain subclasses and display more functional antibody profiles than males, with these differences becoming apparent after puberty [2, 23]. In particular, females often exhibit higher levels of the more inflammatory and functionally potent IgG3 subclass, whereas males more frequently produce higher levels of the less functional IgG4 subclass in response to certain antigens [124]. These patterns suggest that sex hormones, especially estrogens, play a pivotal role in shaping humoral immunity. Positive influences of estrogens on B cell‐dependent humoral immunity in females, across a range of infections and vaccinations, are well documented [2, 125]. Physiological concentrations of estradiol stimulate humoral responses to infection [126], with further enhancement during the periovulatory stage of the menstrual cycle [127], potentially by inducing somatic hypermutation and class switch recombination in B cells through upregulation of activation‐induced deaminase [128]. Moreover, estrogen influences IgG galactosylation by modulating the activity of galactosyltransferase enzymes. This modification improves complement activation and promotes pathogen clearance, thereby contributing to stronger immune responses in females [129, 130]. Galactosylated IgG immune complexes can engage inhibitory FcγRIIB receptors, dampening inflammatory responses such as those mediated by C5aR and CXCR2. This is particularly evident during pregnancy or in remission phases of autoimmune diseases, suggesting that increased galactosylation tempers excessive immune activation [131]. Conversely, testosterone deficiency in men, or the absence of functional ARs in male mice, results in increased numbers of B cells [132]. This effect is partly due to the inhibitory action of androgens on B cell‐activating factor (BAFF), a critical cytokine for B cell activation, differentiation, and survival. Estrogens, in contrast, upregulate BAFF expression, further supporting B cell functions [37].

Altogether, physiological estrogen levels promote more potent and functionally diverse humoral immune responses in females through coordinated effects on B cell activation, antibody subclass distribution, and IgG glycosylation, while androgens act in an opposing, immunoregulatory manner.

#### 4.2.2 CD4 ^+^ and CD8 ^+^ T Cells

CD4^+^ and CD8^+^ T cells are key lymphocytes with complementary roles in immunity that, together, constitute about 30%–60% of PBMCs [133]. CD4^+^ T cells typically constitute around 66% of these T cells, whereas CD8^+^ T cells represent roughly 33% [134]. CD4^+^ T helper (Th) cells coordinate immune responses through specialized subsets. Th1 cells produce IFNγ, IL‐2, and TNF to combat intracellular pathogens, Th2 cells produce IL‐4, IL‐5, and IL‐13 to promote antibody responses and contribute to allergy, Th17 cells produce IL‐17 and IL‐22 to recruit neutrophils during inflammation, and regulatory T cells (Tregs) express FOXP3 to suppress excessive immune activation. CD8^+^ cytotoxic T cells directly kill infected or malignant cells through perforin and granzymes and also secrete cytokines like IFNγ to support broader immune responses [135, 136, 137, 138]. Together, they work to protect the body from infections and diseases, with CD4^+^ cells helping to activate CD8^+^ cells and other immune components. Sex differences exist in both CD4^+^ and CD8^+^ T cell populations, with females typically having a higher CD4:CD8 ratio compared to males, that are more likely to have low/inverted CD4:CD8 ratios, linked to immune aging and inflammation [2, 139, 140]. CD4^+^ and CD8^+^ T cells from females exhibit a higher expression of the early activation marker CD69 [23]. These differences might be influenced by sex hormones, particularly estrogen. Low estrogen levels (such as after menopause) increase CD4^+^ T cell counts [141, 142] while high levels (such as during pregnancy) reduce both CD4^+^ and CD8^+^ T cell numbers [143]. In contrast, androgens tend to suppress lymphopoiesis, mainly affecting T cells during early development when ARs are expressed [144, 145].

#### 4.2.3 CD8 ^+^ T Cells

As early as the activation of CD8^+^ T cells upon antigen presentation, sex differences become evident, with females displaying a higher proportion of activated CD8^+^ T cells than males following in vitro stimulation of human PBMCs [146]. This enhanced response includes greater upregulation of antiviral and proinflammatory effectors, such as increased production of IFNγ, lymphotoxin beta, granzyme A, IL‐12RB2, and granulosyin, while IL‐17A is more highly expressed in males [147]. In adipose mice, females have more activated (CD69^+^) CD8^+^ T cells than males, and their CD8^+^ T cells produce higher levels of IFNγ, TNF, and granzyme B ex vivo [139]. On the single‐cell CD8^+^ T cell level, cells from young adult women show higher expression of the early activation marker *CD69* compared with their male counterparts, along with increased levels of *GNLY*, *CCL5*, *NKG7*, and *IFITM3*—key mediators of the effector phase of CD8^+^ T cell immunity that integrate granule‐dependent cytotoxicity with chemokine‐driven immune recruitment and innate antiviral defense [23]. Functionally, female CD8^+^ T cells have an intrinsic propensity to differentiate into short‐lived effector cells more readily after stimulation, particularly in the presence of IL‐12, while male CD8^+^ T cells tend to form more memory precursor effector cells. This skewing in females is linked to heightened IL‐12 sensitivity, potentially mediated by estrogen effects on CD8^+^ T cells and their IL‐12 receptors [148]. During chronic viral infections, estrogen further promotes CD8^+^ T cell survival and memory formation, enhancing viral clearance in females compared to males [2, 149]. Consistently, female‐derived CD8^+^ T cells often express higher levels of granzyme B and perforin, molecules critical for killing infected cells [125]. In murine models of viral infection, this translates into stronger cytolytic activity and greater expansion of CD8^+^ T cells in females, effects that are diminished after ovariectomy and restored with estrogen [125]. During viral infection, estrogen enhances virus‐specific CD8^+^ T cell responses, including effector molecule expression and cytokine production, whereas testosterone suppresses these functions, shaping sex‐specific memory CD8^+^ T cell differentiation [20, 150].

In summary, females generally mount stronger CD8^+^ effector T cell responses, producing more inflammatory molecules and differentiating faster into short‐lived effector cells, which may contribute to faster pathogen clearance and improved post‐infection outcomes compared to males.

#### 4.2.4 CD4 ^+^ T Cells

CD4^+^ T cells display sex differences in their cytokine profiles, with females exhibiting stronger Th1‐ and Th2‐type immune responses, whereas males show a predominance for Th17 responses [2, 142, 151]. Comparative analyses of PBMCs and CD4^+^ T cells in adult healthy men and women reveal further distinct sex‐associated functional profiles [146]. Under submaximal stimulation with anti‐CD3 and anti‐CD28, female‐derived CD4^+^ T cells exhibit enhanced IFNγ production and proliferative capacity, whereas their male counterparts display a bias toward IL‐17A secretion [152]. Consistent with these findings, subsequent investigations demonstrated that both peripheral CD4^+^ T cells and lamina propria lymphocytes from women express higher levels of IFNγ and the Th1‐associated chemokine CCL5, and contain a greater proportion of proliferating (Ki67^+^) cells, relative to those from men [153]. Similarly, studies in mice show an upregulation of Th1 signature in females including IFNγ [152]. ER signaling appears crucial, as female CD4‐*Cre* × *ERα* ^fl/fl^ CD4^+^ T cells display defective proliferation and T cell activation in response to agonistic antibodies CD3 and CD28 [154]. Exogenous estrogen increases IFNγ production by CD4^+^ T cells in female mice in an ERα‐dependent manner [143].

Nevertheless, there is some conflicting evidence on the direction of Th1/Th2 bias between males and females. Some studies suggest that males display a stronger Th1 profile, with higher IFNγ and IL‐2 and lower IL‐10 and IL‐4, pointing to a relative Th2 bias in females. Other investigations in both humans and mice, however, report fewer Th1 cells in males, implying enhanced Th1 activity in females [155]. Conversely, low estrogen levels favor Th1 immunity by upregulating the IFNγ promoter [156], IRF5 [157], TLRs [158], and T‐bet [159], promoting a Th1 shift with enhanced antigen‐specific CD4^+^ T cell responses [155, 156]. In ovariectomized mice, estrogens also enhance the expression of chemokine receptors (CCR1, CCR5), which contribute to T cell homing [160]. These receptors bind chemokines such as CCL3, CCL4, and CCL5, which mediate the recruitment of immune cells—including monocytes, T cells, and natural killer cells—to sites of inflammation [161]. As estrogen rises, for example during the luteal phase in the menstrual cycle, immunity shifts toward Th2, with increased IL‐4 and IL‐10 and reduced IFNγ [162, 163] via an ERα‐driven increase in GATA‐3 expression [164]. Higher estrogen and progesterone levels, such as during pregnancy, further promote Th2 responses [165] with increased production of IL‐10 and IL‐4, alongside reduced IL‐2 and IL‐17 [166, 167, 168, 169, 170], presumably to support fetal tolerance [155]. Androgens like testosterone can increase the levels of IL‐10 in CD4^+^ lymphocytes via AR signaling [171] and several reports indicate that Th17 immune responses are higher in males [151, 152, 172, 173, 174]. Studies in transgender men undergoing GAHT revealed that testosterone affects CD4^+^ T cell function by inhibiting Th1 and Th17 differentiation via direct AR signaling pathways [175]. Studies in mice and humans indicate that this sexual dimorphism is dependent on androgen status and the T cell expression of peroxisome proliferator activated receptor (PPAR) α and PPARγ. Androgens upregulate PPARα and downregulated PPARγ in human CD4^+^ T cells, shaping pathways of T cell differentiation. PPARα knockdown enhances IFNγ production in male T cells, consistent with a Th1 shift, whereas PPARγ silencing increases IL‐17A production in female T cells, pointing to enhanced Th17 polarization. These findings reveal sex‐specific mechanisms by which androgens modulate CD4^+^ T cell responses [152].

In conclusion, the balance of Th1 and Th2‐type immune responses by CD4^+^ T cells differs between males and females, with substantial evidence for a modulatory role of sex hormones and shifts in immunity as hormones fluctuate during the menstrual cycle.

#### 4.2.5 Regulatory T Cells

Regulatory T cells (Tregs), typically defined as CD4^+^CD25^+^ T cells expressing the transcription factor FOXP3, are central to maintaining immune homeostasis and self‐tolerance [176] and represent 5%–15% of CD4^+^ T cells [177]. They suppress effector T‐cell activity through inhibitory cytokines (IL‐10, TGFβ), IL‐2 consumption, and CTLA‐4‐mediated modulation of antigen‐presenting cells [178]. In infection, Tregs help limit tissue damage caused by excessive inflammation but can also suppress protective immunity, allowing chronic or persistent infection [179]. Men display higher frequencies of CD4^+^CD25^hi^ Tregs compared to women [180]. However, this changes when estrogen levels are high, as more Tregs are found during the follicular phase in the menstrual cycle [181]. The expression of FOXP3 is also higher in males compared to females, a difference shaped by both genetic and hormonal influences. In transgenic male mice, the maternal X chromosome is less methylated, allowing higher expression of the *FoxP3* gene. Because all of their T cells carry the maternal X, this leads to uniformly high FoxP3 protein levels, which protect against autoimmunity. In contrast, female mice have random XCI, so only about half of their T cells express the maternal X. As a result, overall FoxP3 expression is lower, making them more susceptible to autoimmunity [182]. Hormones further modulate this pattern: androgens directly promote FoxP3 expression [183], whereas ERα signaling downregulates it [154]. Similarly, the autoimmune regulator (AIRE) gene, critical for Treg development, is upregulated by testosterone and downregulated by estrogen and progesterone, resulting in higher thymic AIRE expression in males compared to females [184]. Functionally, increased PI3K signaling in males enhances Treg suppressive capabilities [185]. In transgender men undergoing GAHT, six months of testosterone exposure promoted a transcriptional program in naïve CD4^+^ T cells favoring differentiation into Tregs, leading to increased Treg frequencies [175]. Single‐cell RNA sequencing of peripheral PBMCs from healthy cisgender women and men revealed that Treg gene expression is strongly influenced by sex and remains stable with age. Females showed higher activation of pathways related to Th17 differentiation, T cell activation, and IL‐6 production, driven by upregulation of CYBA, CCR4, CD74, and the X‐linked IL‐2RG [23].

Taken together, men have higher Treg frequencies and FoxP3 expression, with enhanced Treg function and suppressive capacity.

### 4.3 Summary of Immune Cells

**FIGURE 1:**
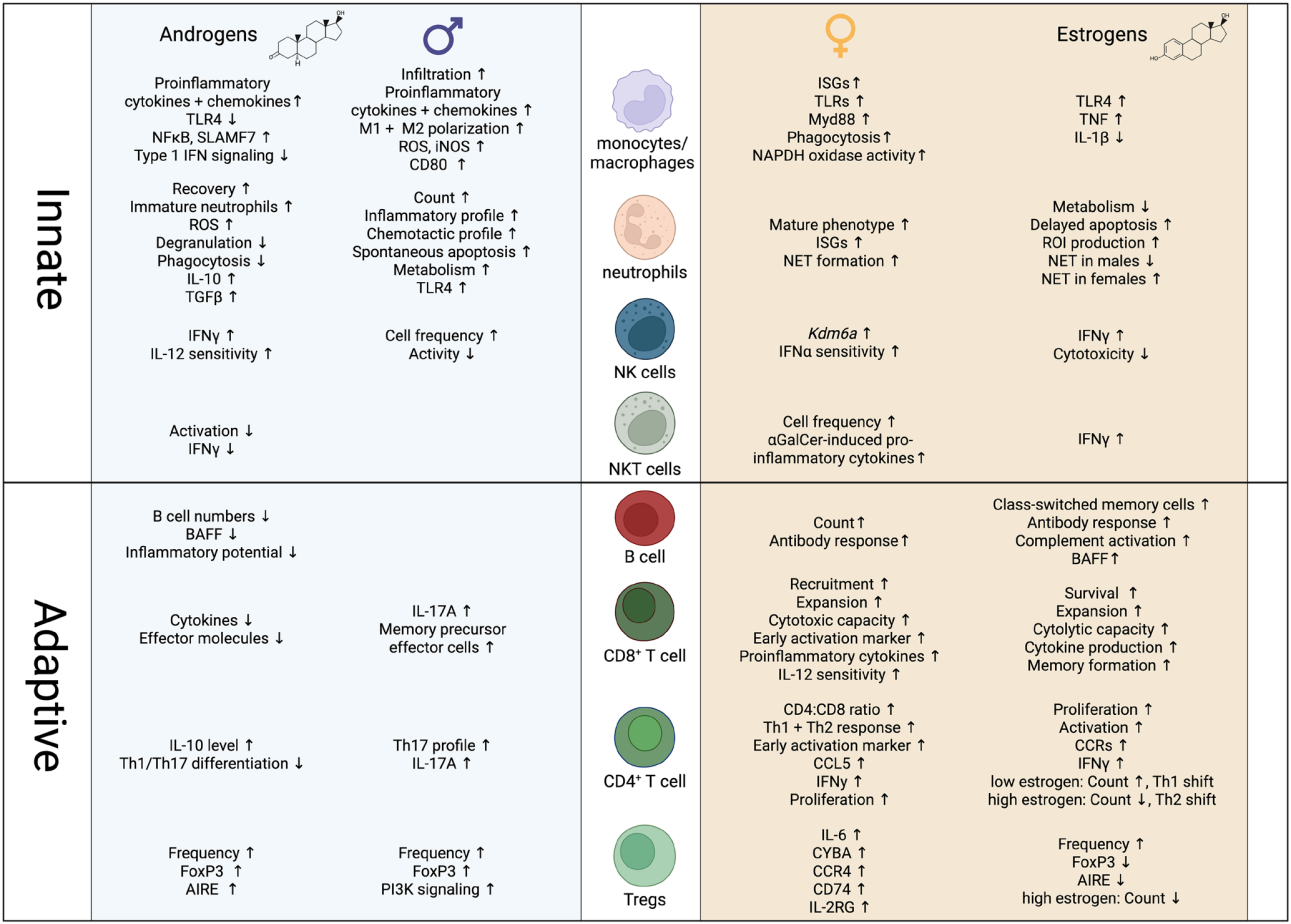
Sex differences across innate and adaptive immune cell types. Multiple innate and adaptive immune cells exhibit sex‐specific differences at steady state and during infection and inflammation. General differences are listed in the columns headed by the male and female symbol. The outer left and right panels illustrate differences with evidence for a modulatory effect of androgens (left) and estrogens (right). Regarding innate immunity, males tend to display higher levels of pro‐inflammatory cytokines as well as increased numbers of monocytes and neutrophils with a less mature phenotype. Females show higher expression of ISGs across several cell types. Within adaptive immune cells, females generally exhibit enhanced B cell activity and stronger Th1 and Th2 responses, whereas males display more pronounced Th17 responses. Abbreviations: AIRE, autoimmune regulator gene; αGalCer, α‐galactosylceramide; BAFF, B cell‐activating factor; CCR, C‐C motif chemokine receptor; FoxP3, forkhead box protein P3; IgG, immunoglobulin G; IFN, interferon; IL, interleukin; iNOS, inducible nitric oxide synthase; LPS, lipopolysaccharide; NET, neutrophil extracellular trap; NFκB, nuclear factor kappa B; ROI, reactive oxygen intermediates; ROS, reactive oxygen species; SLAMF7: SLAM Family member 7; Th, T helper (cell); TLR, toll‐like receptor.

Even under steady‐state conditions, clear sex differences exist in immune cells, shaped by hormonal status and chromosomal factors. Cell‐type specific sex differences described above are summarized in Figure 1. Recent work has highlighted the mechanisms behind overall stronger immune responses in females, showing that pathways such as IFN signaling, complement and coagulation cascades, and IL‐6‐JAK‐STAT3 tend to be more active [21], creating an immune landscape that is more vigilant against infections. Such sex‐specific patterns illustrate how biological differences can shape immune responses, influencing both protection and disease susceptibility.

## 5 Sex Differences in Parasitic Diseases

In the following section, we focus on parasitic diseases, some of which belong to the group of NTDs, including leishmaniasis, Chagas disease, amebiasis, and schistosomiasis [10], as well as malaria. For these diseases, data from both human studies and rodent models provide evidence of biological sex differences that are independent of gender‐related factors (Table 1).

**TABLE 1:** Sex‐specific determinants of parasitic infections in human and rodent models.

Disease	Parasite	Clinical bias in humans	Sex difference in immune response (human)	Bias in rodent model	Role of sex hormones (rodent model)
Cutaneous leishmaniasis (CL)	Old world *Leishmania (L.) major* *Leishmania (L.) tropica* *Leishmania (L.) aethiopica* New world *Leishmania* (*L*.) *mexicana* *Leishmania* (*L*.) *braziliensis* *Leishmania (L.) panamensis* *Leishmania (L.) guyanensis*	M > F Infection rates	M > F IL‐10 and TNF, TLR2 F > M GM‐CSF and IFNγ	M > F IgG1/IgG2; Disease severity (lesion size, parasite burden); Age dependency, higher in adult males; Elevated intralesional IL‐4, IL‐10, TGFβ in males F > M CD4^+^ T cell specific IL‐4Rα‐KO: reduced IgG1, increased healing	T (F): larger lesions, higher parasite load E (F): increased killing of macrophages, NO via PI3Kγ
Visceral leishmaniasis (VL)	*Leishmania (L.) donovani, Leishmania (L.) infantum (formerly L. chagasi)*	M > F More severe disease, Higher parasite burden after puberty	M > F Th2 skewed, IL‐6, IL‐10 and TNF; Increased chemokine expression (mRNA level); Parasite burden in macrophages; IgG1/IgG2 in post–kala‐azar dermal leishmaniasis F > M Th1, IFNγ and IL‐1β; Type I IFN response (mRNA level)	M > F IL‐6, IL‐17, TNF, IL‐10; IL‐17KO protective + associated with neutrophil recruitment; Granuloma formation F > M IL‐12, IL‐1β, IFNγ	T and S (M): Th2 response (IL‐10, IL‐4, IL‐13, TGFβ), increased uptake in bone marrow‐derived macrophages; C (M): reduces parasite burden E: increases Th1; suppresses infection (F + M)
Chagas Disease (CD)	*Trypanosoma (T.) cruzi*	M > F CM, myocardial fibrosis, Apical aneurysms, Parasympathetic nerve cell destruction	M > F Altered amino acid metabolism, Glutamine deficiency F > M CD4^+^/CD8^+^ T cell ratio	M > F Severe myocarditis; Subordinate males exhibiting greater parasite burden F > M Survival	T: exacerbates acute infection severity E: protective
Amebiasis	*Entamoeba (E). histolytica*	M > F Hepatic amebiasis	M > F Serum CCL2 F > M IgG, IgG1; Complement activity; NKT cell‐dependent IFNγ	M > F Larger liver lesions, reduced parasite control; Splenocyte‐specific IL‐4; Serum TNF, CCL2, CXCL1, IL‐23; TNF^+^Ly6C^hi^ monocytes, CXCL1^+^Ly6C^hi^ monocytes; More Th17 (RORγt) cells, more BiTregs; More immature neutrophils; F > M Splenocyte‐specific IFNγ, NKT cell‐dependent IFNγ; FoxP3 on Tregs and BiTregs; CX3CR1 expression on monocytes; Mature neutrophils; Type I IFN response in neutrophils (mRNA level)	T (M): increases Ly6C^hi^ monocyte‐dependent TNF, CCL2, CXCL1 production, upregulation of CD86^+^ expression, downregulation of CX3CR1 MFI on monocytes; C (M): reduces liver pathology; T (F): increases liver pathology, reduces NKT‐ cell dependent IFNγ, increase in monocytes + neutrophils
Malaria	Human *Plasmodium (P.) falciparum* *Plasmodium (P.) vivax* *Plasmodium (P.) malariae* *Plasmodium (P.) ovale* *Plasmodium (P.) knowlesi* Murine *Plasmodium (P.) berghei* *Plasmodium* (*P*.) *chabaudi*	M > F Infection rates, Parasitemia, Mortality	F > M IgM; Faster parasite clearance (asymptomatic infection)	C57BL model: F > M Survival; IgG, IgG1, IL‐10, IFNγ; Higher expression of Th1‐associated genes and type I ISGs CBA/Ca model: M > F Lower mortality	C57BL model: T: loss of infection control; decrease in IgG, IgG1, IgG2b C (F): reduces IgG, IgG1, IL‐10, IFNγ E: induces IFNγ CBA/Ca model: T: decreases parasitemia E: increases parasitemia C (F): increased IFNγ, decreased IL‐10
Schistosomiasis	*Schistosoma* (*S*.) *mansoni* *Schistosoma* (*S*.) *japonicum* *Schistosoma* (*S*.) *haematobium*	M > F Infection rate + intensity; Disease burden; Severe Symmer's fibrosis; Mortality	M > F IL‐13, IgE, IgG1, IgG4 F > M IL‐10, TGFβ, IFNγ; Circulating Tfh cells, ICOS^+^ and PD‐1^+^ Tfh; Stronger inflammatory response (mRNA level)	F > M Disease burden, worm burden, mortality	T (M): higher T correlates with lower worm burden T (M + F): protective during early phase of infection E: protective in urogenital infection

Abbreviations: BiTreg, bifunctional regulatory T cell; C, castration; CCL, C‐C motif chemokine ligand; CCR, C‐C motif chemokine receptor; CL, cutaneous leishmaniasis; CM, cardiomyopathy; CX3CR1, C‐X3‐C motif chemokine receptor 1; E, estradiol (treatment); F, female; FoxP3, forkhead box protein P3; GM‐CSF, granulocyte‐macrophage colony‐stimulating factor; ICOS, inducible co‐stimulator; IFN, interferon; Ig, immunoglobulin; IL, interleukin; KO, knockout; M, male; NKT, natural killer T; NO, nitric oxide; PD‐1, programmed cell death protein 1; PI3Kγ, *phosphoinositide 3‐kinase* gamma; RORγt, retinoic acid‐related orphan receptor gamma t; S, (hormone) substitution; T, testosterone (treatment); Tfh, T follicular helper (cell); TGFβ, transforming growth factor beta; Th, T helper (cell); TLR, toll‐like receptor; TNF, tumor necrosis factor; VL, visceral leishmaniasis.

### 5.1 Trypanosomatida

Trypanosomatids belong to the class kinetoplastida and are a diverse group of unicellular flagellates characterized by a unique organelle, the kinetoplast. This organelle comprises a dense network of mitochondrial DNA situated near the base of the parasite's flagellum [186]. There are three major species that are transmitted by hematophagous insects in a zoonotic or anthroponotic life cycle which are responsible for severe human diseases: *Leishmania* (*L*.) spp., the causative agents of leishmaniasis; *Trypanosoma* (*T*.) *cruzi*, the etiological agent of Chagas disease; and *T. brucei* , the causative agent of African trypanosomiasis (also called sleeping sickness) [187, 188, 189]. Except for sleeping sickness, these diseases are considered among the most significant NTDs, affecting over 27 million people worldwide and causing approximately 150,000 deaths annually [9, 187, 190]. Due to the fortunately drastic decrease in sleeping sickness cases over recent years (from over 30,000 to fewer than 1000 annually) [191], no data exist on sex differences in prevalence or symptoms, leading us to exclude the disease from the review.

### 5.2 Leishmaniasis

Leishmaniases are infectious diseases caused by intracellular *Leishmania* parasites, transmitted through the bite of female sandflies (*Phlebotomus* spp. (Old world); *Lutzomyia* spp. (New World)). About 20 species are pathogenic to humans, together affecting more than 12 million people in 98 tropical and subtropical countries. Each year, an estimated 600,000‐1000,000 new cases of cutaneous leishmaniasis (CL) and 50,000–90,000 cases of visceral leishmaniasis (VL) occur [192]. The clinical spectrum ranges from self‐limiting skin ulcers in CL (e.g., *L. major*, *L. tropica*), to severe mucosal destruction in mucocutaneous leishmaniasis (mainly *L. braziliensis*), and systemic VL (primarily *L. donovani* and *L. infantum*) [193], and is governed by the parasite and the host immune response. There are increasing numbers of reports on sex difference in the outcome of both, CL and VL, with higher incidence and disease severity in males [194, 195, 196, 197, 198, 199, 200, 201, 202]. The sex difference in both VL and CL is more pronounced in adults compared with children [203, 204], suggesting that sex hormones play a role in influencing disease outcome. Several of these studies have proven that the observed differences between males and females persist when infection rates are similar between the sexes, revealing this to be a biological effect rather than the result of different gendered behavior [203, 204, 205].

At the sandfly bite site, myeloid cells such as neutrophils and monocytes rapidly infiltrate the tissue and are preferential targets of the parasite. In case of systemic VL, classical monocytes distribute the parasite to the spleen and the liver [206, 207]. Neutrophils can internalize *Leishmania* parasites and either contribute to host protection [208] or promote parasite survival [209]. In the latter case, macrophages engulf parasite‐laden apoptotic neutrophils, enabling the parasites to enter these cells as a “Trojan horse” and facilitating their transfer into macrophages [210]. Once infected, macrophages become the main host cells, and their polarization determines disease outcome. M1 macrophage polarization, driven by Th1‐derived IL‐12 and IFNγ, results in production of nitric oxide (NO) and pro‐inflammatory cytokines that enhance parasite clearance, while Th2‐driven M2 macrophage polarization leads to secretion of IL‐10 and TGFβ, suppressed immunity, and long‐term parasite persistence [207, 211, 212]. DCs capture parasites and prime T cells in lymph nodes [213]. Effector T cells mediate immediate outcomes, while memory T cells sustain long‐term protection [190, 207, 214].

However, despite growing insights into the immune response to *Leishmania* infection, data on sex‐based differences in human immunity remain very limited. In a selected cohort of patients with localized CL caused by *L. mexicana* , 90% were male and only 10% female. Notably, significantly higher serum levels of GM‐CSF and IFNγ were associated with resistance in females [195]. In vitro studies using human moMΦs infected with *L. infantum* revealed higher infection rates and parasite loads in macrophages from men compared with those from women [215]. Regardless of sex, infection induced upregulation of IL‐10, IL‐8, and TNF, alongside downregulation of CCL2 and IL‐18 in the supernatant of infected cells. At the transcriptomic level, macrophages from women showed a strong upregulation of type I IFN (IFNα/β) signaling and proinflammatory cytokines, with increased expression of ISGs (e.g., *IFIT2*, *IFIT3*, *IFIT5*, *OASL*) as early as 6 h post‐infection. In contrast, the reduced resistance of macrophages from males was linked to higher expression of chemokines such as *CXCL1*, *CXCL3*, *CCL7*, and *CCL20*. This profile favors the recruitment of innate immune cells, particularly neutrophils, which may facilitate increased parasite uptake at the infection site in vivo [215]. Moreover, estradiol treatment slightly reduced parasite burden, but this effect was observed only in macrophages from women [215]. Generally, type I IFNs are well recognized for their role in promoting antiviral and antitumor immunity by supporting protective Th1 polarization [63]. However, their role in chronic VL caused by *L. donovani* appears to differ. In patients with VL, IFNα and IFNβ were identified as key negative upstream regulators of CD4^+^ T cells, acting through accessory T‐cell functions that mediate IL‐10 production, thereby suppressing protective Th1 responses [216]. These findings were supported by studies in *Ifnar1*‐deficient mice, which display resistance to *L. donovani* infection [216]. Such seemingly contradictory results may be explained by fundamental differences between acute and chronic infection stages: type I IFN stimulation prior to, but not during, *Leishmania* infection confers resistance in mice [216].

It is generally assumed that females mount stronger antibody responses to infectious challenges, including vaccines, although notable exceptions exist [49]. In leishmaniasis, despite numerous epidemiological studies describing sex differences in CL and VL, data on sex‐specific stratification of serum immunoglobulin titers remain limited. Elevated *Leishmania*‐specific IgG has been proposed as a marker for progression from asymptomatic to symptomatic VL [217] or mucocutaneous leishmaniasis [218]. Overall, sex differences in antibody titers are not consistent across studies. Where humoral data are available, they are often not comparable between sexes or confounded by disease stage and exposure history. One study reported sex‐specific analyses of anti‐*Leishmania* IgG titers in two independent VL cohorts from India and Sudan [219]. Among Indian patients over 16 years of age, males exhibited higher IgG titers than females, whereas in Sudanese patients, females showed higher titers. However, these differences were not statistically significant [219]. Similarly, a study from Colombia found no sex differences in anti‐*Leishmania* IgG titers among CL patients [220]. A more recent study of patients in India with post‐kala‐azar dermal leishmaniasis—a complication of VL that occurs predominantly in males, but not during VL itself—found a correlation of higher anti‐leishmanial IgG titers in males with higher serum testosterone levels [221]. Experimental work in mice has provided more detailed insights. In *L. mexicana* infection, male mice displayed higher IgG1 and IgG2a titers despite exhibiting greater disease severity compared with females [222]. Under defined immunological conditions, when CD4^+^ T‐cell IL‐4Rα signaling was disrupted, female mice infected with *L. donovani* showed significantly reduced antigen‐specific IgG1 production compared with males. These findings suggest sex‐dependent differences in antibody isotype responses, with male mice developing a stronger Th2‐associated IgG1 response during infection compared to female mice despite the lack of IL‐4Rα [223]. Further investigations on sex differences in immune responses in leishmaniasis models largely depend on the *Leishmania* species as well as the rodent or hamster strain. Cutaneous infection of hamsters with *L. (Viannia) panamensis* or *L. (Viannia) guyanensis* results in chronic lesions typical of the human disease caused by these parasites. Within this model, sex hormones may influence disease outcome: prepubertal male animals develop smaller and less severe lesions than adult males, suggesting that puberty and rising androgen levels increase susceptibility [220]. Consistently, testosterone treatment of female animals leads to significantly larger lesions compared with untreated females [220]. The greater disease severity observed in males is associated with elevated intralesional expression of IL‐4, IL‐10, and TGFβ, with *TGFβ* mRNA levels strongly correlating with lesion size, pointing to a more permissive, disease‐promoting immune response in males [220]. Further studies in mice revealed higher serum levels of TNF and IL‐10 in male (BALB/c; C57BL/6) animals, along with increased IL‐6 and IL‐17 production by splenocytes following *L. infantum* infection. In contrast, females exhibit increased expression of proinflammatory cytokines IL‐1β and IFNγ in splenocytes with no significant difference in IL‐4 levels [205]. During *L. donovani* infection, male IL‐6‐ and IL‐17A‐deficient mice show improved outcomes, including lower parasite burdens in spleen and liver, enhanced CD4^+^ T cell‐dependent IFNγ production, and reduced neutrophil recruitment [206, 224]. Given the tendency toward stronger Th17 responses in males and the specific role of IL‐17 in leishmaniasis [225], Lockard et al. propose that elevated IL‐6 may enhance Th17 differentiation, establishing an IL‐6‐dependent Th17 pathway that contributes to a sex‐specific immunopathological axis in VL [201]. Sex hormones also directly affect parasite survival. DHT promotes *L. mexicana* promastigote development in BMDMs, leading to enhanced parasite replication and increased infection rates [226]. Similarly, hamster models of *L. donovani* infection show that males are more susceptible and harbor higher parasite burdens compared to females. Exogenous testosterone exacerbates disease in both male and female animals, whereas estradiol suppresses infection. Castration of males reduces parasite levels, while ovariectomy in females promotes disease, supporting the protective role of female sex hormones and the exacerbating effect of male hormones [227]. Mechanistically, testosterone inhibits apoptosis of *L. donovani* ‐infected BMDMs, leading to increased parasite uptake and survival [227, 228], potentially via modulation of the p38 MAPK pathway, although direct inhibition of this pathway by testosterone has not been observed [229]. In contrast, estradiol enhances macrophage NO‐dependent killing through a PI3Kγ‐mediated pathway in rodents [230, 231].

Overall, sex differences in leishmaniasis influence both innate and adaptive immunity, with stronger evidence for effects on macrophages and T cell responses than on antibody production. Women generally exhibit more robust type I IFN‐driven proinflammatory programs in macrophages, whereas men display chemokine profiles that may enhance parasite uptake, with outcomes shaped by infection stage and context‐dependent IFN activity. Sex‐specific differences in antibody responses are less consistent in humans, though mouse studies suggest females may be less prone to Th2‐associated IgG1 responses, indicating isotype‐specific regulation. These differences reflect the interplay of hormones, immune mechanisms, and parasite factors: androgens tend to favor parasite survival, while estradiol promotes control. Future human studies should systematically investigate sex‐biased immune responses in leishmaniasis, and experimental animal models that reproduce human sex differences remain invaluable for dissecting these mechanisms.

### 5.3 Chagas Disease

Chagas disease (CD), caused by the protozoan parasite *T. cruzi* , is primarily transmitted by the feces of blood‐sucking *Triatominae* bugs. It remains a major public health concern, with 6–7 million cases and about 10,000 deaths annually, mostly in Latin America [232]. CD is also increasingly reported in Europe, North America, Japan, and Australia, where transmission occurs mainly through blood transfusion, organ transplantation, and vertical routes [233, 234].

After entering through skin fissures or mucosal surfaces, trypomastigotes invade host cells, replicate as amastigotes, and disseminate as bloodstream trypomastigotes [235]. Acute infection is often asymptomatic or associated with mild systemic symptoms [232, 236]. Chronic infection develops when parasites persist intracellularly [237], and about 30% of patients later develop cardiac or gastrointestinal complications, including megaesophagus, megacolon, and chronic Chagas cardiomyopathy [232, 238, 239].

The immune response in the acute phase is characterized by the recognition of *T. cruzi* molecules such as glycosylphosphatidylinositol (GPI)‐mucins, glycoinositolphospholipids (GIPLs), and DNA by TLR2, TLR4, and TLR9 on innate immune cells, activating NFκB and triggering cytokines like IL‐12, TNF, and IFNγ [240, 241, 242]. These cytokines activate NK cells and macrophages, while chemokines such as CCL2, CCL3, CCL4, CCL5, CXCL9, and CXCL10 recruit leukocytes to infection sites and are extremely important in the control of acute infection [243], but exacerbated expression may maintain the inflammation leading to myocardial tissue damage [244, 245]. Adaptive immunity follows, with cytotoxic CD8^+^ and CD4^+^ T cells producing IFNγ, Th17 cells modulating inflammation, and antibodies supporting parasite control [246, 247], while a Th2 profile with IL‐4 is associated with susceptibility [248]. Infection is usually controlled within 2–3 months, however, the parasite can evade immunity using specific pathogenicity factors that block antibody or T‐cell activity [249, 250, 251] as well as chemokine dysregulation, which can contribute to chronic disease and tissue damage [245]. In chronic CD, a strong Th1‐pro‐inflammatory response with high IFNγ, TNF, and IL‐6 drives cardiac inflammation [252, 253] and likely enhances CD8^+^ T cell activity, contributing to both parasite control and tissue damage. In contrast, the indeterminate form features IL‐10, Treg, and Th17 responses that regulate inflammation allowing CD8^+^ T cells to control the parasite without harming tissue [254, 255, 256]. The balance between pro‐ and anti‐inflammatory signals determines disease severity and chronic outcomes [245].

Evidence for sex differences in CD in humans is limited but points to important patterns. While *T. cruzi* infection rates are similar between sexes in endemic rural areas, progression to CD and particularly Chagas cardiomyopathy (CM) is more common in men [257, 258]. Necropsy studies show that men with chronic CD are more likely to develop apical aneurysms, suggesting sex‐specific cardiac involvement linked to parasympathetic nerve cell destruction [259]. Clinical studies also report greater myocardial fibrosis, adverse ventricular remodeling, and lower ejection fraction in male patients, indicating more severe structural and functional cardiac damage [260, 261]. Underlying mechanisms may involve sex hormones and immune modulation. Several studies found either higher male incidence [262, 263] or no significant sex differences [264], while biomarker analyses reported no clear sex bias in cardiac markers across disease stages [265]. However, male CD patients exhibit altered amino acid metabolism, notably glutamine deficiency, which may relate to elevated sex hormone levels [266]. Immunophenotyping further suggests stronger adaptive responses in females, with higher CD4:CD8 T cell ratios compared to males [267]. These differences may help explain why men more frequently progress to severe cardiac outcomes [260, 268]. In addition, a retrospective study from Spain emphasized overlooked infections in asymptomatic women of reproductive age, highlighting the need for improved screening [269, 270]. These discrepancies may reflect underdiagnosis, regional variability, or the difficulty of attributing nonspecific cardiac manifestations to CD [271, 272, 273].

Experimental models provide additional insight into sex‐based differences in CD progression. In F344 rats, both sexes are susceptible to infection, but only females survive [274]. In mice, sex strongly modulates disease outcomes across genetic backgrounds. Male C57BL/6 and C3H/HeN mice develop more severe myocarditis than females, though outcomes vary with *T. cruzi* strain [275]. In BALB/c mice, males show higher parasitemia and mortality, and testosterone exacerbates acute infection severity, whereas estradiol is protective [276, 277, 278]. Social status within the mice groups further influences outcomes, with subordinate males exhibiting greater parasite burdens, likely due to stress‐induced susceptibility [276].

Together, these findings indicate that sex plays a critical role in shaping CD progression, with men showing a higher risk for cardiac complications in both clinical and experimental contexts. However, contradictory results in humans underscore the need for better surveillance, sex‐specific profiling, and integration of mechanistic insights from animal models.

### 5.4 Amebiasis

Invasive amebiasis is caused by intestinal infection with the protozoan parasite *Entamoeba (E.) histolytica*. Humans are the only natural hosts, acquiring infection through ingestion of *E. histolytica* cysts in contaminated food or water [279]. The disease is endemic in tropical and subtropical regions worldwide, particularly in Central and South America, Asia, and Africa, with especially high prevalence reported in Bangladesh, India, Brazil, Colombia, Mexico, and China [280, 281]. Globally, amebiasis accounts for more than 25,000 deaths annually [282] and contributes to an estimated 2.2 million disability‐adjusted life years (DALYs) [283].

Following infection, trophozoites emerge from cysts and invade the intestinal mucosa, resulting in severe hemorrhagic amebic colitis. When dissemination occurs via the bloodstream, trophozoites may form abscesses in various organs, most commonly the liver [284]. Notably, although infection rates are equal between the sexes or higher in women, hepatic amebiasis—also referred to as amebic liver abscess (ALA) – shows a marked increase after puberty and peaks in adult males [285, 286]. A male bias in the incidence of amebic colitis is reported in some studies as well [285, 287], but less distinct and less well characterized than in hepatic amebiasis.

The higher resistance of women to the development of hepatic amebiasis appears to involve several key factors. During dissemination from the intestine to the liver, trophozoites are exposed to complement activation. Because of their high sensitivity to complement, most trophozoites are destroyed, and only a few survive by developing resistance through the expression of serine/threonine protein kinases [288]. Notably, serum from women is significantly more effective at killing *E. histolytica* trophozoites than serum from men, and this effect is complement‐dependent [289]. In addition, women develop higher total IgG and especially IgG1 levels during asymptomatic infection compared to men [290]. IgG1, a strong activator of complement with the longest half‐life among IgG subclasses, further enhances complement‐mediated killing [291]. Other protective innate immune mechanisms include the NO sensitivity of *E. histolytica* trophozoites. Both in vitro and in vivo studies have shown that IFNγ‐dependent induction of NO by neutrophils or macrophages exerts potent amebicidal activity, leading to efficient parasite control [292, 293, 294].

In the murine model of hepatic amebiasis, which mirrors the sex‐related differences observed in humans, splenocytes from female mice produced higher levels of IFNγ [295]. Importantly, in the liver, NKT cells represent an early and major source of IFNγ [102]. In this model, disease severity increases in the absence of NKT cells, whereas treatment with the specific ligand αGalCer in the murine model reduces the liver pathology after infection [108, 296]. Moreover, NKT cells from women produce more IFNγ following ex vivo stimulation with amebic antigens compared to NKT cells from men [110]. Similarly, in the murine model, activated NKT cells from female mice generate more IFNγ than those from males. This enhanced IFNγ production in female‐derived NKT cells is inhibited by testosterone substitution, while orchiectomy increases IFNγ production in male‐derived NKT cells. Consequently, liver damage decreases in castrated male mice but increases in female mice receiving testosterone substitution [118].

Interestingly, in the serum of men asymptomatically infected with *E. histolytica* , among a variety of measured pro‐ and anti‐inflammatory cytokines, only the chemokine ligand CCL2 was found at significantly higher levels [290]. CCL2, along with other members of the CCL family, plays a critical role in recruiting innate immune cells such as monocytes and neutrophils [297]. In murine models, higher frequencies of hepatic Ly6C^hi^ monocytes and neutrophils are observed following infection, contributing to liver destruction through immunopathological mechanisms [58, 81]. Accordingly, depletion of monocytes and neutrophils using specific antibodies, or genetic ablation of the CCL2 receptor in CCR2^−/−^ mice, markedly reduces liver destruction in male mice, whereas adoptive transfer of monocytes increases abscess size [298]. As demonstrated in respective knockout mutant mice, the immunopathological IL‐23/IL‐17 axis [299] underlies these innate immune mechanisms. IL‐23, produced by cells such as macrophages and DCs, drives the differentiation and maintenance of IL‐17‐producing Th17 cells, thereby creating a pro‐inflammatory environment. This pathway recruits and activates monocytes and neutrophils at sites of infection, contributing to abscess formation and liver tissue damage [300]. Additionally, it enhances the production of other pro‐inflammatory cytokines, including TNF, IL‐6, and IL‐1, further amplifying the inflammatory response and exacerbating liver pathology [58, 296, 301, 302]. At the same time, IL‐23 inhibits IL‐13‐dependent tissue repair mediated by regenerative Ly6C^lo^ monocytes [300].

Serum and plasma levels of CCL2, CXCL1, TNF, and IL‐23 are higher in male mice during ALA compared to females [58, 81]. Moreover, Ly6C^hi^ classical monocytes from male mice produce higher levels of intracellular CCL2, CXCL1, and TNF compared to female mice during hepatic amebiasis, and these responses are further amplified by testosterone treatment [58]. Presence of CX3CR1, which mediates monocyte retention in the bone marrow and adhesion to endothelial cells [303], is higher on Ly6C^hi^ monocytes from female mice and decreased by testosterone treatment of gonadectomized male mice [58]. TNF, presumably produced also by Kupffer cells, plays a central role in the immunopathology of this model, as depletion of this cytokine or depletion of Kupffer cells by clodronate treatment substantially reduces liver destruction [298]. Supporting this observation, human monocytes from trans men receiving testosterone treatment showed elevated secretion of CXCL1 and TNF following ex vivo TLR4 stimulation with LPS [58]. Not only Ly6C^hi^ monocytes, but also neutrophil numbers are higher in male compared with female naïve and *E. histolytica* ‐infected mice in multiple organs, such as the bone marrow, the liver, and blood [58, 81]. Gonadectomy decreases neutrophil numbers in the liver and the blood, while testosterone treatment increases them in male and female mice, independent of infection [81]. The increased numbers of neutrophils in male mice during ALA are associated with a lesser mature phenotype compared to females [81]. Similarly, ex vivo stimulation of isolated bone marrow‐derived neutrophils with LPS increases expression of CD54, a marker for proinflammatory (N1) neutrophils and PD‐L1, a prototypic marker for immunosuppressive neutrophils (N2) in both sexes [304]. Transcriptomic analysis of neutrophils provides insight into these sex‐specific differences [81]. Neutrophils from females exhibit higher expression of classical type I ISGs under steady‐state conditions in both bone marrow and blood (e.g., *Oas2*, *Ifih1*, *Rsad2*), whereas a distinct subset of ISGs is highly regulated in male counterparts (Bone marrow: *Ddit4*, *Slc25a28*, *Trim30b*, *Irf1*, *Jade2*; Blood: *Ssbp3*). During infection, more ISGs are upregulated in female blood neutrophils (ALA vs. naïve) compared with male samples, in which ISGs are generally downregulated. Type II ISGs in bone marrow neutrophils show less pronounced sex differences under steady‐state conditions. However, chemokines involved in neutrophil adhesion and recruitment are expressed at higher levels in male‐derived neutrophils. At the protein level, the prototypical type I antiviral effector RSAD2 (Radical S‐adenosyl methionine domain‐containing protein 2), also known as viperin [305], is more highly expressed in neutrophils from female mice. Notably, this expression was independent of testosterone treatment [81].

The sex‐specific differences observed in innate immune responses are also present in the adaptive immune compartment during ALA. Liver infection by *E. histolytica* activates hypoxia‐inducible factor 1α (HIF‐1α) in the surrounding hepatic tissue, which influences the recruitment of Th17 cells and Tregs [302]. As shown above, Th17 cells play a pivotal role in the immunopathology of hepatic amebiasis via induction of CCL2 [300]. Notably, male C57BL/6 mice harbor more retinoic acid‐related orphan receptor gamma t (RORγt^+^) Th17 cells, more FoxP3^+^ Tregs and more RORγt^+^ bifunctional Tregs (BiTregs) [306] in the naïve liver compared to female mice, but only the sex difference in RORγt^+^ Th17 cells and RORγt^+^ BiTregs persists during infection [302]. However, the expression of the immunosuppressive transcription factor FoxP3 is significantly higher in BiTregs and FoxP3^+^ Tregs of female mice [302], thereby mitigating the severe immunopathology observed in males [296]. Together, these findings suggest a coordinated interplay between innate monocyte and neutrophil responses and adaptive Th17/Treg regulation that shapes the sex differences in liver pathology during ALA.

Taken together, female hosts are able to control liver pathology caused by *E. histolytica* infection at early stages through protective IFNγ‐mediated immunity. In contrast, in the murine model, males develop severe immunopathology driven by an excessive innate immune response characterized by elevated monocyte recruitment and the initiation of an immunopathological Th17/IL‐23 immune axis. Evidence from human cohorts suggests similar mechanisms in humans.

### 5.5 Schistosomiasis

Schistosomiasis is caused by infection with *Schistosoma* trematodes and is endemic to parts of Middle and South America, Asia, and Africa, where the vast majority of transmissions occur [307, 308, 309]. In 2021, around 151 million cases of schistosomiasis occurred, resulting in over 1.7 million DALYs and over 12,800 deaths [309, 310]. Prevalence, intensity of infection, disease burden, and mortality from schistosomiasis are consistently higher in males compared with females across different age groups in children, adolescents, and adults [31, 309, 310, 311, 312].

Three main groups of *Schistosoma* (*S*.) cause disease in humans, namely *S. japonicum* (East Asia), *S. haematobium* (West Asia and Africa), and *S. mansoni* (Middle and South America, Africa) [307]. *Schistosoma* cercariae are released by freshwater snails as the intermediate hosts and infect humans by penetrating their skin, which may result in contact dermatitis [307]. In the human host, cercariae mature into schistosomulae, migrate along the vasculature via the heart and lungs, and finally develop into adult worms. Male and female adult worms mate in the hepatic portal vein, which leads to the deposit of highly antigenic and cytotoxic eggs in several organs as the worms migrate, mainly the urogenital tract, intestine, and liver [307, 313]. These eggs are the primary cause of pathology during *Schistosoma* infection as the result of tissue inflammation during granuloma formation around the egg, driven by the host immune response [314]. Clinical manifestations of urogenital schistosomiasis caused by *S. haematobium* include genital pain and hematuria, and may result in renal failure, infertility, and bladder cancer [307, 315]. Infection with *S. mansoni* and *S. japonicum* predominantly affects the intestine and liver, resulting in abdominal pain, diarrhea, hepatomegaly, among other symptoms [307, 315]. Acute infection with *S. japonicum* is often accompanied by fever (‘Katayama fever’), but is rarely reported in infection with other species [315].

The immune response during the first weeks of infection is predominantly a Th1 type immune response, involving the release of cytokines such as IL‐12 and IFNγ [313, 316]. Production of eggs by the female worm, which starts around 5–6 weeks after infection, triggers a shift to a Th2 response and increased release of IL‐4, IL‐5, and IL‐13, as well as isotype switching to IgE [313, 314, 316]. Infiltration of eosinophils, T cells, B cells, macrophages, and neutrophils results in the formation of a granuloma surrounding the egg, protecting the surrounding tissue from excessive inflammation induced by egg toxins, with slight variations in immune cell composition between different tissues and *Schistosoma* species [313, 314, 316]. However, granuloma formation also promotes excessive wound healing, thus leading to fibrosis. This process is crucially mediated by T cell‐derived IL‐13, which stimulates collagen production by fibroblasts [314, 317, 318]. IL‐13 furthermore promotes M2 macrophage polarization, resulting in increased Arginase 1 activity and thus production of proline, required for collagen synthesis [316]. In the liver, hepatic stellate cells additionally contribute to collagen production [319]. Severe periportal fibrosis (also called Symmer's fibrosis) may lead to obstruction of blood flow, splenomegaly, portal hypertension, and eventually bleeding, and therefore constitutes one of the main causes of morbidity and mortality in schistosomiasis [313].

In individuals with Symmer's fibrosis following *S. mansoni* infection, men and women show distinct patterns of fibrosis severity according to age. A study in Sudanese individuals detected lower prevalence of no or low fibrosis scores and higher prevalence of moderate and advanced fibrosis in adolescent and adult men compared to women, but no sex differences in children [320]. Similarly, while adolescent and young adult Brazilian females exhibit predominantly low severity scores of fibrosis and severity is increased in older women, all severity stages of fibrosis are present in men of all age groups, suggesting an earlier onset of severe fibrosis in men [321]. In the Brazilian study, severe fibrosis was associated with significantly elevated serum IL‐4, IL‐5, and TNF in men, but not in women. Interestingly, serum IL‐13 was decreased in severe fibrosis patients compared to individuals without fibrosis, but still elevated in men with fibrosis compared with women [321]. Conversely, whole blood of fibrosis patients stimulated ex vivo with *Schistosoma* soluble egg antigen revealed decreased IL‐6, IL‐10, and TNF and elevated IL‐13 in severe hepatic fibrosis compared with nonfibrotic individuals, with IL‐13 again significantly higher in males than females [322]. As described above, IL‐4, IL‐5, and particularly IL‐13 are associated with the shift to Th2 immunity during granuloma formation and the promotion of fibrosis, and higher levels of these cytokines in males may therefore contribute to higher disease burden.

The cytokines IL‐10, TGFβ and IFNγ are associated with protection from severe fibrosis by regulating inflammation [322, 323, 324]. In chronically *S. haematobium*‐infected adults with low intensities of infection, stimulation of PBMCs ex vivo with adult worm antigen (AWA) resulted in higher secretion of TGFβ and IL‐10 by female‐derived cells, but lower secretion of Th1‐associated TNF and IFNγ [325]. In addition, IgA levels were higher in sera from females compared to males in this study, with no sex differences in other antibody classes [325]. Both IgA and TGFβ production were previously shown to be mediated by estrogens in different contexts [326, 327], hence, female sex hormones may also play a role in modulating their production during schistosomiasis. Nonetheless, other studies report no sex difference or higher IgA in males in *S. mansoni* and *S. japonicum* infections [311, 328]. Furthermore, multiple studies comparing antibody responses to AWA from both *S. mansoni* and *S. japonicum* in sera from infected individuals showed higher IgG1, IgG4 and IgE in male adolescents and adults in cohorts from Kenya, the Philippines, Brazil, Uganda and China [311, 328, 329, 330, 331].

*Schistosoma* infection is associated with elevated circulating levels of follicular T helper (Tfh) cells [332, 333]. In females infected with *S. japonicum* , total Tfh cells as well as Tfh subsets expressing inducible co‐stimulator (ICOS) and programmed cell death protein 1 (PD‐1) are higher in females than in males [332]. Tfh cells play a dual role in *Schistosoma* infection, as they are important for antibody production but may also contribute to liver pathology [333, 334]. ICOS and PD‐1 on Tfh cells act as co‐stimulatory molecules in the interaction with B cells, promoting their differentiation into plasma cells and antibody production [335, 336].

The role of antibodies in schistosomiasis remains to be fully elucidated, with several reports showing immunopathological effects particularly for IgG4, whereas high levels of IgE are proposed to protect from severe disease and IgA production may be both protective and pathological [331, 337, 338, 339]. To interpret the abovementioned observations, further research is needed on how antibody levels, protection or disease progression during schistosomiasis, and biological sex intersect.

On a transcriptional level, *S. haematobium* infection leads to stronger inflammatory responses in female‐derived peripheral immune cells compared to their male counterparts as evidenced by a higher magnitude of gene regulation, although it was not specified which genes were regulated [340].

In *Schistosoma*‐infected men, serum testosterone levels are significantly lower than in non‐infected individuals [341]. This may be due to an interference of the inflammatory response during granuloma formation with steroidogenesis in the testes [341], as macrophage‐derived pro‐inflammatory cytokines like TNF and IL‐6 have been shown to reduce testosterone production by Leydig cells [342]. Also in male C57BL/6 mice, infection with *S. japonicum* leads to a reduction in serum testosterone levels and, conversely, castrated male mice have higher worm burdens [343]. However, AR blocking by treatment of mice with flutamide does not alter worm burdens [343]. Testosterone can bind *Schistosoma* glutathione S‐transferase and block its enzymatic activity, essential for parasite survival and reproduction [344], hence, testosterone may exert a protective role during infection by directly acting on the parasite. Similarly, in CBA/J mice infected with *S. mansoni* , females and castrated males exhibit lower survival rates and higher worm burdens than untreated males or females and castrated males treated with testosterone [345]. This protective effect of testosterone is only observed when treating mice prior to or in early phases of infection, not after 5 weeks at the start of egg production. Considering the female bias in *Schistosome* infection‐related morbidity and mortality in these mice [345, 346], findings from this model should be inferred to infection in humans with caution. Evidence for a protective role of estradiol comes from another study, in which injection of *S. haematobium* eggs into the bladder of female mice treated with tamoxifen and male mice resulted in more tissue damage than in control female mice [347].

Endemic regions for schistosomiasis, particularly on the African continent, are to a large extent also characterized by a high prevalence of human immunodeficiency virus (HIV) infection, resulting in significant numbers of co‐infections [348]. Several studies have reported that infection with *Schistosoma* species resulted in an elevated risk of HIV infection and HIV‐related death particularly in women, but not in men [348, 349, 350, 351]. Elevated inflammation and altered immune cell populations in the genital mucosa during female genital schistosomiasis, including more HIV target cells such as CD4^+^ T cells, may be involved in the higher risk of HIV, but detailed mechanisms behind sex differences in these co‐infections have not been studied yet [348]. Additionally, individuals with HIV co‐infections excrete fewer schistosome eggs, reducing transmission of schistosomiasis, whereas HIV transmission is elevated in both sexes [348, 352, 353].

Collectively, men suffer from higher schistosomiasis burdens, possibly due to increased pro‐fibrotic immune responses during granuloma formation, whereas immune responses associated with protection from severe fibrosis are enhanced in women. The role of sex hormones in the context of *Schistosoma* infection remains unclear, as both testosterone and estradiol may have protective properties.

### 5.6 Malaria

Malaria is caused by protozoan parasites of the genus *Plasmodium*, which are transmitted by the bite of female *Anopheles* mosquitoes [354]. Five different *Plasmodium (P.)* species are known to naturally cause malaria in humans [355], with *P. falciparum* causing the most severe form of the disease. In humans, *Plasmodium* parasites undergo a complex life cycle. After infection, they first infiltrate the liver and replicate in hepatocytes before emerging into the bloodstream to infect circulating erythrocytes, in which they undergo differentiation in multiple stages [354]. Upon completed stage differentiation, infective merozoites burst from erythrocytes in a synchronized manner, resulting in the malaria‐characteristic repeated fever attacks patients experience with every new erythrocytic cycle of the parasite [356].

In 2023, 263 million malaria cases and an estimated 597,000 deaths occurred worldwide [357], making malaria the parasitic infection with the highest global disease burden. 94% of all malaria cases and 95% of deaths, 76% of which are children under the age of 5, occur in the disproportionately highly affected WHO African region [357].

Many epidemiological studies report higher case numbers among males compared with females [358, 359, 360, 361, 362, 363, 364]. In the Americas, both malaria incidence and deaths are higher among men and boys, whereas studies from Ghana and Uganda report more cases among females [365, 366]. However, disease severity does not differ between the sexes in the latter and this female bias is likely the result of differences in healthcare‐seeking behavior [365]. Assessment of overall differences in malaria rates between men and women is complicated by the fact that only half of all endemic countries in the highly affected African region reported gender‐disaggregated data in 2023 [357]. Insights into biological sex differences stem from studies assessing not only malaria prevalence but also clinical factors. In reports from Sudan, Ghana, and Uganda, parasitemia was found to be lower and survival rates higher in females compared to their male counterparts [358, 367, 368]. In asymptomatically infected individuals with similar infection rates in a Ugandan cohort, females were found to clear parasites faster than males [369]. In contrast, a study from northeast India showed higher malaria incidence, severity, and higher anemia among females over the age of 15 [370].

Furthermore, women are uniquely at risk of one of the most severe forms of the disease—placental malaria during pregnancy [371]. In the WHO African region, 34% of all pregnancies were affected by maternal malaria infection in 2023 [357], constituting a major health issue for women and their offspring. Children from mothers who experienced placental malaria have a higher risk of malaria disease during infancy due to long‐term immune alterations caused by in utero exposure to *Plasmodium* antigens, with male children more at risk than female children [372, 373].

The male bias reported in many studies is most pronounced in adolescents and adults, indicating a role of sex hormones in the modulation of disease outcome [358, 360, 367, 369]. Additionally, in response to vaccination, several studies have reported higher anti‐malarial antibody titers in females, particularly in post‐pubertal individuals [374, 375]. The steroid hormone dehydroepiandrosterone sulfate (DHEAS), a precursor of sex hormones essential during pubertal maturation, was found to correlate positively with enhanced resistance to *P. falciparum* infection during puberty in both girls and boys [376, 377]. Since both sexes were studied separately, it is unclear whether the protective effect of DHEAS differs in magnitude between males and females.

Already in 1980, the male bias in malaria was proposed to be the result of higher acquired immunity in females, as evidenced by higher IgM titers in females over the age of 5 [358]. In spite of a lack of human studies, insights into immune responses and the role of sex hormones in the context of *Plasmodium* infection come from mouse models, which replicate the male bias observed in humans.

During the liver stage of *Plasmodium* infection, the immune response is primarily mediated by CD8^+^ T cells and a Th1 type cytokine profile contributing to parasite elimination, wherein IFNγ plays a key role [356]. In *P. chabaudi*‐infected C57BL/6 mice, females exhibit lower mortality and faster recovery than males [378, 379, 380]. They produce more total IgG and IgG1 antibodies as well as the cytokines IL‐10 and IFNγ—an effect that is ablated by gonadectomy of female mice and induced by estradiol treatment [379, 380]. Expression levels of Th1 immune genes (including *Ccl3*, *Cxcl10*, *Ccr5*, and several type I ISGs) as well as genes regulating T cell activation (like *Tnfrsrf4*) are higher in blood leukocytes of female mice than males at peak parasitemia and are modulated by gonadectomy [379]. In mouse strains lacking B and/or T cells (TCRβδ^−/−^, μMT, RAG1), the male bias is even more pronounced than in C57BL/6 mice, with males suffering from more severe symptoms such as anemia and weight loss, while exhibiting higher parasitemia than their female counterparts [379]. In IFNγ^−/−^ C57BL/6 mice, the sex difference in mortality is mitigated and loss of IFNγ is more detrimental to female mice [379]. In addition, testosterone treatment of C57BL/6 and C57BL/10 mice results in loss of infection control, accompanied by a decrease in total IgG, IgG1, and IgG2b antibody titers, suppressed hepatic expression of immunoregulatory genes, and impaired response to vaccination [381, 382, 383, 384, 385]. Chemical inhibition of the AR showed that the immunosuppressive effect exerted by testosterone was AR‐independent [386]. Curiously, treatment of C57BL/10 mice with low levels of estradiol, in contrast to the higher physiological levels used in the abovementioned studies, also led to immunosuppression, likely through the ER [387].

In a vaccination study, higher IFNγ production by hepatic CD8^+^ T cells led to increased protection from challenge with *P. berghei* infection in adult female mice [388]. This difference between the sexes was not present in juvenile mice and was found to be modulated by an inhibitory effect of testosterone on the adaptive immune response [388, 389]. No changes were observed upon removal of estrogens in gonadectomized female mice [388, 389]. Both male and female mice form efficient CD8^+^ T cell memory responses following vaccination, but the presence of androgens, particularly at the time of challenge, reduced the cytolytic capacity of these cells, as characterized by lower granzyme B production, as well as impaired recruitment of further CD8^+^ T cells to the liver [389].

Contrasting findings come from a CBA/Ca model infected with *P. berghei*. In this model, parasitemia is higher in female mice and treatment with estradiol increases, whereas testosterone decreases parasitemia [390, 391]. Gonadectomy of female mice led to increased IFNγ and decreased IL‐10 levels [391] and gonadectomized male mice had lower IgG than intact males in one study, but another showed the opposite effect under testosterone treatment [390, 391]. Interestingly, gonadectomy of both sexes resulted in reduced TNF levels [391]. Testosterone treatment of gonadectomized mice of both sexes resulted in increased IFNγ, but also increased IL‐10, alongside decreased IL‐6 [390]. In infected, gonadectomized male and female mice, testosterone enhanced levels of CD3^+^ T cells and NK cells, and dampened B220^+^ B cell levels [390]. In CBA mice, *P. berghei* parasitemia is decreased by estradiol, but cerebral malaria severity and mortality is heightened [392], while in Balb/c mice, this hormone is protective and enhances survival [393]. These findings show that the role of sex hormones in modulating immunity during murine malaria is complex and depends highly on the mouse and parasite strain used, exposing the limitations of animal studies for understanding human malaria.

In summary, the disease burden of malaria is biased toward males, likely due to an inhibition of protective T cell‐mediated immunity by testosterone, at least during the liver stage of the disease. Considering the lack of data regarding sex differences from human studies alongside inconsistent findings from mouse models, further research is needed to understand the underlying differences in immune processes between males and females.

## 6 Conclusion

Across multiple parasitic infections, males generally experience a higher disease burden than females, reflecting fundamental sex differences in immune function. Although evidence from human studies is still limited, rodent models have provided important mechanistic insights, particularly into the immunomodulatory roles of sex hormones and their interactions with innate and adaptive pathways.

**FIGURE 2:**
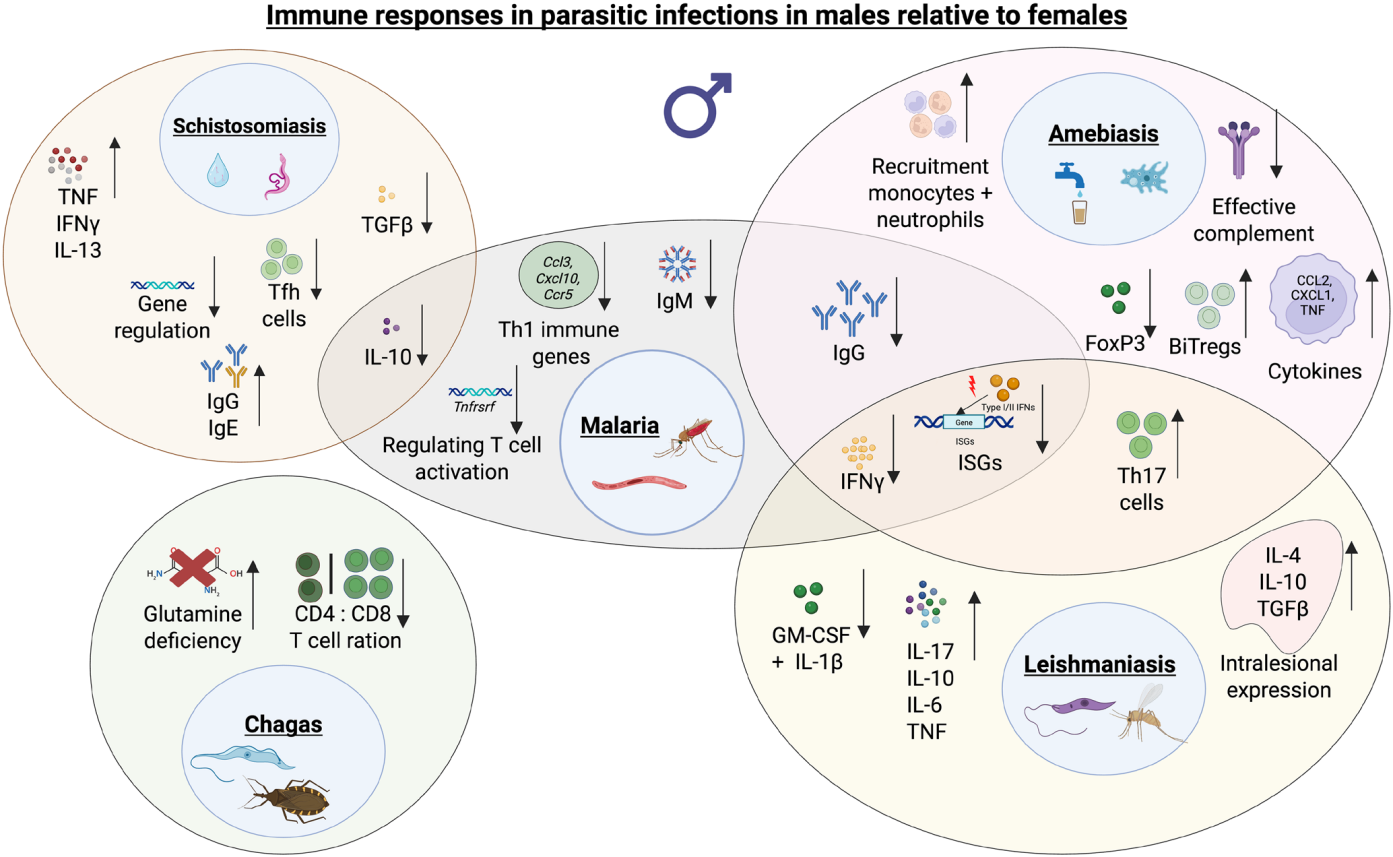
Immune responses in parasitic infections in males relative to females. In males, immune responses differ from those in females across parasitic diseases such as amebiasis, leishmaniasis, malaria, schistosomiasis, and Chagas disease. Although the nature and extent of these differences vary, several patterns are conserved. In this figure, arrows indicate how immune parameters in males vary relative to females for each disease. Males often show lower IgG levels (malaria, amebiasis), lower ISG expression and IFNγ responses (amebiasis, leishmaniasis, malaria), but higher Th17 frequencies (amebiasis, leishmaniasis) than females. In contrast, females show stronger IL‐10 responses (schistosomiasis, malaria). Evidence is most robust for amebiasis, leishmaniasis, malaria, and schistosomiasis, whereas data for Chagas disease remain limited. BiTregs, bifunctional regulatory T cells; CXCL1, C‐X‐C motif chemokine ligand 1; CCL2, C‐C motif chemokine ligand 2; FoxP3, forkhead box protein P3; GM‐CSF, granulocyte‐macrophage colony‐stimulating factor; Ig, immunoglobulin; IFN, interferon; ISGs, interferon‐stimulated genes; IL, interleukin; Tfh, T follicular helper (cell); TGFβ, transforming growth factor beta; Th, T helper (cell); TNF, tumor necrosis factor.

Current data suggest that male‐biased susceptibility arises from the combination of genetic differences and hormonally mediated effects that can amplify pathological responses. While each parasitic infection induces a distinct immune program, several recurring sex‐biased patterns have emerged: males tend to exhibit heightened Th17‐mediated activity in amebiasis and leishmaniasis, whereas females mount stronger IFNγ and IFN‐mediated responses and show higher ISG expression across amebiasis, leishmaniasis, and malaria, along with increased IL‐10 production in malaria and schistosomiasis (Figure 2). Importantly, these shared features do not have uniform consequences; depending on the pathogen and the stage of disease, the same immune pathway can be protective in one context and pathological in another. This context dependency remains one of the major challenges in understanding sex differences in parasitic immunity.

Overall, sex differences in immune function, which are already evident at steady state, persist and diversify during parasitic infection, adding further complexity to the host‐parasite relationship. This review shows that immune responses cannot be generalized across parasites, tissues, or sexes. To fully unravel the mechanisms underlying these sex biases, especially in understudied parasitic diseases, future research should more systemically integrate sex and gender as essential variables in experimental design, data interpretation, and translational efforts.

## Funding

This work was supported by Deutsche Forschungsgemeinschaft, LO1426/4‐1.

## Conflicts of Interest

The authors declare no conflicts of interest.

## Data Availability

Data sharing is not applicable to this article as no datasets were generated or analysed during the current study.
